# Outcomes used in randomised controlled trials of nutrition in the critically ill: a systematic review

**DOI:** 10.1186/s13054-018-2303-7

**Published:** 2019-01-14

**Authors:** Garry Taverny, Thomas Lescot, Emmanuel Pardo, Frederique Thonon, Manar Maarouf, Corinne Alberti

**Affiliations:** 1 Paris Diderot University, Sorbonne Paris-Cité, INSERM U1123, Paris, France; 2 Unit of Clinical Epidemiology, Assistance Publique-Hôpitaux de Paris, CHU Robert Debré, Paris, France; 3 Department of Anesthesiology and Critical Care Medicine, Saint-Antoine Hospital, Assistance Publique-Hôpitaux de Paris, Paris, France; 4 Sorbonne Université, UPMC Univ Paris 06, Paris, France; 5 EA 7334 REMES (Recherche Clinique ville hôpital, Méthodologies et Sociétés), Paris Diderot University, Paris, France

**Keywords:** Intensive care unit, Clinical nutrition, Outcome measure, Randomized controlled trials

## Abstract

**Background:**

No evidence exists to date on which to base the selection of outcome measures for assessing nutritional interventions in critically ill patients. We conducted a systematic literature review to describe the outcomes used in recent randomised controlled trials (RCTs) assessing nutritional interventions in critically ill patients. Our objective was to set the foundation for the development of a core set of outcome measures for use in future RCTs.

**Methods:**

We searched the PubMed/MEDLINE, Cochrane Central Register of Controlled Trials, and ClinicalTrials.gov databases for RCTs of nutritional interventions in critically ill patients aged 18 years or older, published and/or registered between January 2000 and August 2018. Outcomes were divided into six categories (mortality, length of stay, duration of organ dysfunction, complications, functional outcomes, and others) and analysed according to the study characteristics and publication year.

**Results:**

Of the 885 references retrieved, 170 were included in the review. Of these, 136 (80%) defined a primary outcome, 114 (67%) defined secondary outcomes (two per study on average), and 34 (20%) did not specify whether outcomes were primary or secondary. We identified 24 different outcomes in all, of which 19 were primary. Complications were the most widely used primary outcome (65/136, 48%). Mortality was the primary outcome in 17/136 (13%) studies, with six different timepoints. The main secondary outcomes were length of stay (90/114, 79%), mortality (82/114, 72%), and duration of organ dysfunction (75/114, 65%).

**Conclusions:**

This systematic review highlights the heterogeneity of outcomes used in recent randomized controlled trials evaluating nutritional interventions in critically ill patients. The results of our systematic review may have implications for designing future RCTs of nutritional interventions in the ICU.

**Electronic supplementary material:**

The online version of this article (10.1186/s13054-018-2303-7) contains supplementary material, which is available to authorized users.

## Background

Patients managed in the intensive care unit (ICU) must receive nutritional support to ensure that their energy and nutrient needs are met. There is evidence that ICU-acquired malnutrition is associated with worse patient outcomes [1]. In observational studies, providing adequate amounts of calories and/or protein is associated with decreased mortality [2, 3]. However, large randomised controlled trials (RCTs) aimed at determining the best timing, energy target, and route of administration of nutritional support either found no efficacy or produced apparently contradictory results [4–9]. These discrepancies are an obstacle to developing consensual clinical nutrition strategies, and to adherence of physicians to clinical guidelines. One likely source of discrepancies is the variability in the outcomes selected to assess the efficacy of nutritional interventions in RCTs. This variability hinders the interpretation of results, comparisons of RCTs, and the development of strong evidence-based recommendations.

In theory, the benefits of adequate nutrition in critically ill patients should include lower risk of acquired infections including wound infections, pressure sores, and muscle mass loss; shorter duration of mechanical ventilation; shorter ICU and hospital stay; and perhaps lower mortality. Nevertheless, no accurate and robust data are available on the benefits of adequate nutrition or adverse effects of inadequate nutrition in critically ill patients. To obtain such data, the optimal outcome for assessing nutritional interventions must be determined. This is a challenging task, as shown by the broad range of outcomes used in RCTs. For example, the mortality rate is often used as the primary outcome in RCTs performed in critically ill patients, including those evaluating nutritional interventions. However, variability in responses to the intervention across patient subsets, with lower mortality in some subsets and higher mortality in others, may produce a net result of no effect. Although the ultimate goal of improving the management of critically ill patients is to improve survival several weeks after the ICU stay, whether the likelihood of achieving this goal can be increased by a short-term nutritional intervention is debatable. Developing a structured, clinically relevant, consensual, and validated RCT methodology may help to better define primary and/or secondary outcomes reflecting improvements in patient outcomes related to nutritional interventions for use in future RCTs.

The objective of this study was to obtain a clear picture of the outcomes used in published RCTs of nutritional interventions in the ICU, as a first step towards selecting optimal outcomes. To this end, we conducted a systematic review of the recent literature.

## Methods

We systematically reviewed recently published RCTs evaluating nutritional interventions in critically ill patients, with special attention to the outcomes used. The complete review protocol was submitted to PROSPERO and the international prospective register of systematic reviews (CRD42016036575), and the review was guided by the Preferred reporting items for systematic reviews and meta-analyses (PRISMA) statement [10].

### Search strategy and article selection

Relevant RCTs published between January 2000 and August 2018 were identified by electronically searching PubMed/MEDLINE (1 January 2000–31 August 2018) and the Cochrane Central Register of Controlled Trials (CENTRAL) (2000–2018). The following MeSH and free text terms were used: “nutrition”, “feeding”, “protein-calorie”, “nutritional support”, “critical care”, “critically ill”, “critical illness”, “ICU”, “adult”, “randomized controlled trial”, “controlled clinical trial”, “randomized”, and “trial”. The search was limited to articles written in English or French. The reference lists of retrieved articles were screened for additional relevant articles. Unpublished relevant RCTs were identified by searching the ClinicalTrials.gov registry using the following criteria: adult (18–65) and senior (66+); from 1 January 2000 to 31 August 2018; completed or terminated or recruiting or not yet recruiting; interventional studies (clinical trials); and the key words nutrition, feeding, protein-calorie, nutritional support, critical care, critically ill, intensive care, ICU, critical illness, and randomized. The search strategy is detailed in the online-only supplement (Additional file 1).

**Fig. 1:**
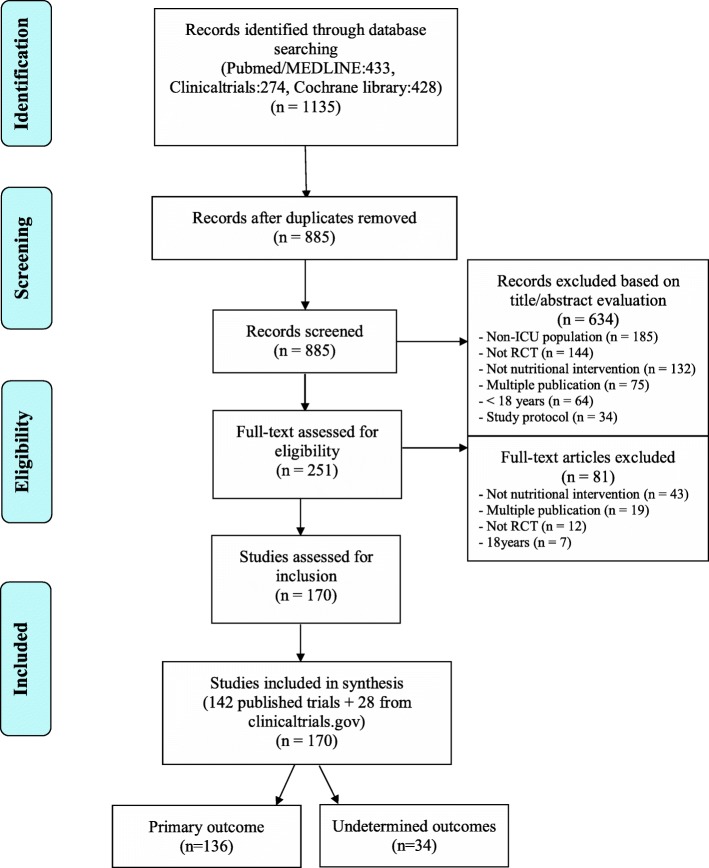
Flow chart showing the phases of the systematic literature review. RCT, randomised controlled trial

Among articles retrieved using the aforementioned search strategy, we selected those meeting the following criteria: RCT, including critically ill adults (age > 18 years), evaluating a nutritional intervention, and published after 1999. We excluded neonatal or paediatric intensive care studies, studies focussing primarily on the pharmaceutical properties of enteral nutrition (EN) or parenteral nutrition (PN) without supplying details on nutritional support, abstracts, case reports, review articles, and ancillary studies. Retrieved articles were first assessed by one of us (GT), who read the titles and abstracts to identify relevant trials. When the title and abstract were considered potentially relevant, the full text was retrieved by two of us (GT and MM), who worked independently of each other to apply the aforementioned criteria for inclusion in our study. Figure 1 is the flow chart of the retrieved and included articles.

### Data collection

For each study, the data were extracted by two of us (GT and MM, EP, or FT), who worked independently of each other. The data were recorded on a standardised form that was pre-tested by one of us (GT) on ten randomly selected articles and modified as needed. The following data were recorded: study characteristics (population, study design, blinding method, source of funding, number of centres, and geographic region); main study topic, recorded as clinical nutrition strategy, composition of nutritional support, or nutritional supplementation (see examples in Table 2); and primary and secondary outcomes. Outcomes were classified using predefined categories including mortality (at any time, ICU mortality, in-hospital mortality, day-28 mortality, day-90 mortality), length of stay (in the ICU and in hospital), duration of organ dysfunction (times on mechanical ventilation, catecholamines, renal replacement therapy; organ failures; and antibiotic therapy), ICU-associated complications (infections, metabolic complications, feeding intolerance), functional outcomes during the study period (muscle strength/walking distance tests, quality of life, physical function), and other (metabolic concentration, feeding measures, tube placement success rate, tube placement time, contamination of packs and glass bottles). Outcomes that were not described as primary or secondary were classified as undetermined. All recorded data were cross-checked after two of us read each study independently of each other. Discrepancies were resolved by discussion between the two readers or by adjudication by another of us (TL) if a consensus could not be reached. Data were entered into the study database using EpiData Software (EpiData Association, Odense, Denmark).

### Definitions

Metabolic concentration was defined as any outcome relevant to the blood level of a substance (e.g., a protein). Short-term and/or long-term metabolic complications were recorded as related to EN or PN. Infections were defined according to the International Sepsis Forum definition of infections in the ICU [11]. Feeding intolerance was defined as a complication due to the administration of nutritional preparations, such as diarrhoea or gastrointestinal symptoms. Feeding measures were all outcomes directly related to the nutritional intervention, such as feed volume and gastric residual volume. Physical function refers to any self-reported capabilities or physical performance. The capabilities assessed included upper limb function (dexterity), lower limb function (walking or mobility), spinal function, and instrumental activities of daily living.

### Risk of bias assessment

The methodological quality of each RCT was assessed using the Cochrane risk of bias tool as indicated in chapter 8 of the Cochrane Handbook [12]. Methodological quality domains were those in the risk-of-bias table, i.e., randomisation and method of randomisation; allocation concealment; blinding of participants, healthcare providers, or outcome assessors; proportion of participants who did not complete follow up; partial reporting of outcomes; and any other areas of potential bias. The risk of bias in each RCT was classified as low, high, or unclear. RCTs were classified as having an overall high risk of bias if the risk of bias was high for at least one domain and low if the risk of bias was low for all domains.

### Statistical analysis

The statistical analysis was performed using SAS version 9.4 (SAS Institute Inc., Cary, NC, USA). Qualitative variables were described as numbers and percentages and quantitative variables as mean and standard deviation if normally distributed and median (25th–75th percentiles) otherwise. Ranges are also reported.

## Results

### Article selection

The initial electronic search identified 1135 articles. No additional articles were identified from other sources. Among the articles found on ClinicalTrials.gov, 250 duplicates were removed. Of the 885 remaining articles, 634 were excluded based on the title and abstract and 81 based on the full text (Fig. 1), leaving 170 RCTs for the study analysis (see Additional file 2).

### Characteristics of selected RCTs

Among the 170 selected RCTs, 142 (84%) were published in 36 different journals and 28 (16%) were registered on ClinicalTrials.gov. The median date of publication was 2010 (2004–2013), and most RCTs (*n* = 89, 52%) were conducted in Europe. Table 1 reports the main characteristics of the selected RCTs. Published trials collectively randomised 30,003 patients (median, 78 (37–157)). Only 54 (32%) RCTs involved multiple centres, with a median of 11 (4–18). The duration of the intervention was reported in 107/170 (63%) RCTs; median duration was 7 (5–14) days.

**Table 1:** Characteristics of the 170 included randomised controlled trials (RCTs); 142 published trials and 28 protocols registered on ClinicalTrial.gov

Characteristics (/170 RCT)	
Population, 143 RCTs, *n* (%)	
Medical	21 (15)
Surgical	34 (23)
Both	88 (62)
Design, 170 RCTs, *n* (%)	
Parallel-group RCT	155 (91)
Cross-over RCT	7 (4)
Factorial-designed RCT	5 (3)
Cluster-designed RCT	3 (2)
Blinding, 170 RCTs, *n* (%)	
None	68 (40)
Blinding^a^	102 (60)
of the patients	88 (86)
of the staff	78 (76)
of the outcome assessors	88 (86)
Funding, 170 RCTs, *n* (%)	
Industry	22 (13)
Public	61 (36)
Both	48 (28)
Not reported	39 (23)
Recruitment, 167 RCTs, *n* (%)	
Single-centre	113 (68)
Multicentre	54 (32)
National	44 (81)
International	10 (19)
Geographic region^b^, 167 RCTs, *n* (%)	
Europe	89 (53)
America	48 (29)
Oceania	20 (12)
Asia	17 (10)
Number of randomised patients per RCT and per year, median [25th–75th percentiles]	
2000 (*n* = 183 patients)	30 [20–37]
2005 (*n* = 836 patients)	53 [40–60]
2010 (*n* = 1087 patients)	43 [32–104]
2015 (*n* = 378 patients)	88 [62–127]
2018 (*n* = 2534 patients)	1267 [124–2410]

^a^The sum of the percentages exceeds 100 because some RCTs had several forms of blinding (blinding of the patient, staff, and assessors)

^b^The sum of the percentages exceeds 100 because some RCTs were conducted in more than one geographic region

Table 2 depicts the distribution of the various topics of the selected RCTs. The most common topic category was clinical nutrition strategy (69/170, 40%), with strategies to optimise delivery and minimise the risk associated with EN being the most common example (*n* = 36). Clinical nutrition composition was the second most common category (66/170, 39%), followed by clinical nutrition supplementation (35/170, 21%).

**Table 2:** Distribution of the main topic categories in the 170 randomised controlled trials (RCTs); 142 published trials and 28 protocols registered on ClinicalTrial.gov

Topics	Number of RCTs (%) *n* = 170
Clinical nutrition strategy	69 (40)
Strategies to optimise delivery and minimise risk of EN	36
Early versus delayed nutrient intake	8
Amount of EN prescribed	14
EN versus PN	7
Strategies to optimise delivery and minimise risk of PN	3
PN versus standard care	1
Composition of clinical nutrition	66 (39)
Composition of EN	30
Composition of nutrition	21
Composition of PN	15
Supplementation in clinical nutrition	35 (21)
Supplemental glutamine	23
Supplemental antioxidants	8
Supplemental PN	3
Vitamins	1

*EN* enteral nutrition, *PN* parenteral nutrition

### Outcomes (Table 3)

The 170 RCTs used 24 different outcomes, which we classified into six categories: mortality, length of stay, duration of organ dysfunction, complications, functional outcomes, and other. A single primary outcome was described in 136/170 (80%) RCTs and at least one secondary outcome in 114/170 (67%) RCTs (two per study on average), whereas 34/170 (20%) RCTs did not specify whether the outcome was primary or secondary; we designated these unspecified outcomes “undetermined outcomes”.

**Table 3:** Description of the primary, secondary, and undetermined outcomes used in randomised controlled trials (RCTs)

Outcome	Primary^a^ *n* = 136/170	Secondary^a^ *n* = 114/170	Undetermined^a^ *n* = 34/170
Mortality, *n* studies (%)	n = 17/136 (13)	82/114 (72)	22/34 (65)
ICU mortality	2	46	17
Hospital mortality	4	44	13
Day-28 mortality	5	20	4
Day-29 to day-89 mortality	4	5	1
Day-90 mortality	2	7	0
>Day-90 mortality	0	15	6
Length of stay, *n* studies (%)	4/136 (3)	90/114 (79)	21/34 (62)
ICU length of stay	4	84	21
Hospital length of stay	0	61	9
Duration of organ dysfunction, *n* studies (%)	12/136 (9)	75/114 (66)	14/34 (41)
Mechanical ventilation	6	56	10
Catecholamine infusion	0	5	1
Renal replacement therapy	0	13	0
Organ failure	6	35	6
Antibiotic therapy	0	13	1
Complications, *n* studies (%)	65/136 (48)	74/114 (65)	30/34 (88)
Infections	27	48	21
Metabolic	19	24	12
Feeding intolerance	19	24	9
Functional outcome, *n* studies (%)	5/136 (4)	12/114 (11)	1/34 (3)
Quality of life	1	7	0
Physical function^b^	2	6	1
Muscle strength	1	6	0
Walking distance	1	2	0
Others, *n* studies (%)	33/136 (24)	16/114 (14)	12/34 (35)
Metabolic concentration^c^	12	13	7
Feeding measures^d^	17	3	4
Tube placement (time needed or success)	3	0	0
Contamination of packs and glass bottles	1	0	0

*ICU* intensive care unit

^a^Some studies had more than one secondary and/or undetermined outcomes

^b^Physical function was assessed based on self-reported capabilities rather than observed physical performance. The capabilities assessed included upper limb function (dexterity), lower limb function (walking or mobility), spinal function, and instrumental activities of daily living

^c^Metabolic concentration was defined as any outcome relevant to the blood level of a substance (e.g., a protein)

^d^Feeding measures were all outcomes directly related to the nutritional intervention, such as feed volume and gastric residual volume

**Fig. 2:**
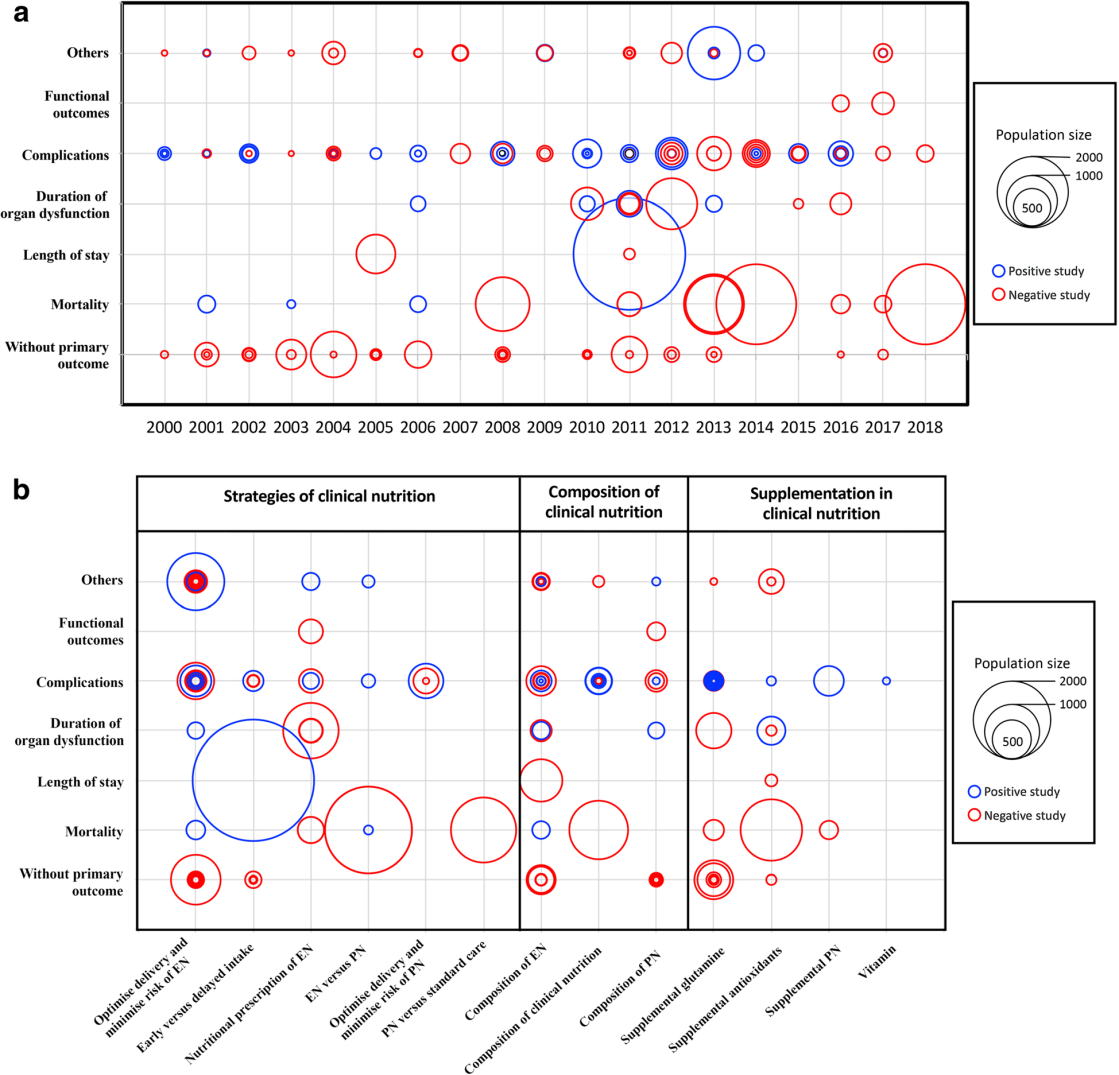
Randomised controlled trials (RCTs) of nutritional support in critically ill patients. The data are from the 142 published RCTs, as the information on the 28 RCTs registered on ClinicalTrials.gov was inadequate. Positive RCTs are those showing a statistically significant difference for the primary outcome; other RCTs are described as negative. **a** Shows the RCTs, with their size, according to type of primary outcomes used from 2000 to 2018. **b** Maps the studies according to population size and nature of the nutritional interventions

**Fig. 3:**
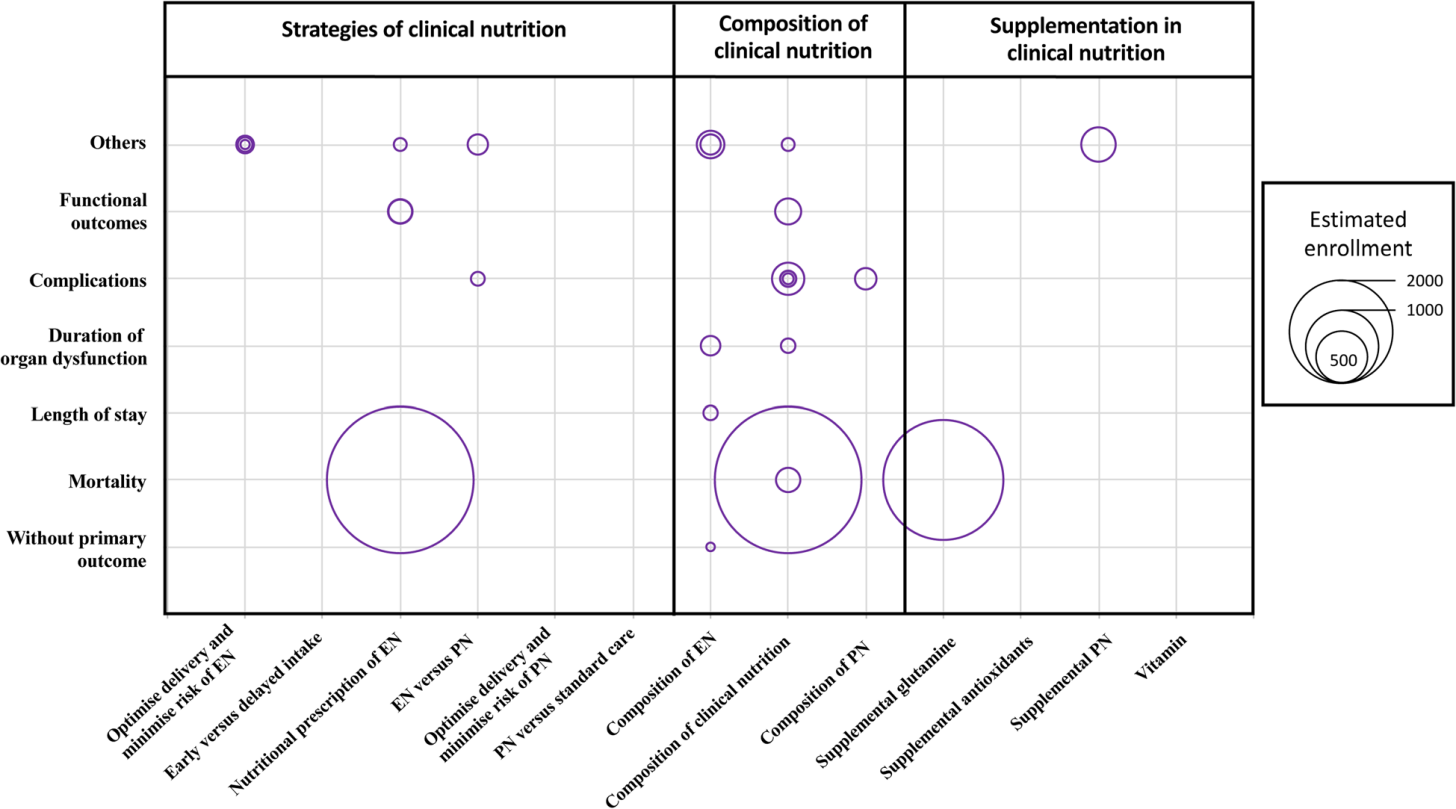
Primary outcomes in 28 protocols of future or ongoing randomised controlled trials registered on ClinicalTrials.gov, according to population size and nature of the nutritional intervention. EN, enteral nutrition; PN, parental nutrition

Of the 136/170 RCTs with one primary outcome, complications represented the most common primary outcome (65/136, 48%). Mortality was the primary outcome in 17/136 (13%) RCTs. Six different mortality time points were used: in-ICU mortality, in-hospital mortality, day-28 mortality, mortality between day 29 and day 89, day-90 mortality, and mortality after day 90. The most common secondary outcomes were length of stay (90/114, 79%), mortality (82/114, 72%), duration of organ dysfunction (75/114, 66%) and complications (74/114, 65%). The primary outcome was functional in 2 (2/136, 1%) of the published trials and 3 (3/136, 2%) of the ongoing or future trials. Figure 2a illustrates the number of published studies by year from 2000 to 2018 according to primary outcome category, number of included patients, and result of the primary outcome. Complications and mortality were the most widely used defined primary outcome. Most of the trials with complications as the primary outcome included fewer than 500 patients, whereas most of the large trials used mortality as the primary outcome and found no significant effect of the tested nutritional strategy. Figure 2b depicts the distribution of topic categories by primary outcome according to the number of included patients. The studies including the largest numbers of patients mainly used length of stay and mortality, with clinical nutrition strategy as the topic. The use of complications as the outcome was fairly well balanced across topic categories. Figure 3 shows the primary outcomes in 28 protocols of future or ongoing (i.e., unpublished) RCTs registered on ClinicalTrials.gov, according to population size and nature of the nutritional intervention, with the goal of predicting the forthcoming results. Mortality is the primary outcome in the largest studies.

### Risk of bias in the selected RCTs

**Fig. 4:**
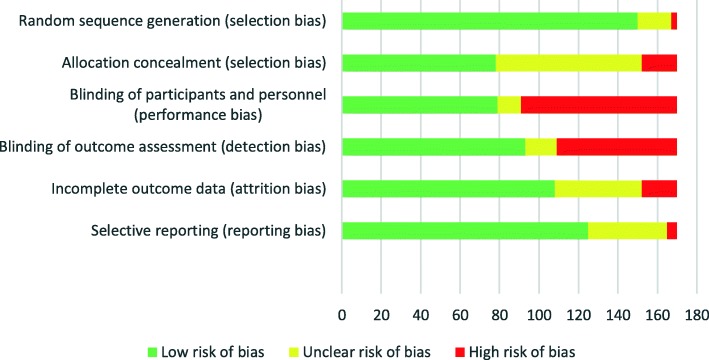
Risk of bias in randomised controlled trials of nutritional interventions in critically ill patients published between 2000 and 2018. We used the Cochrane risk of bias tool to assess the risk of bias for the included trials

Figure 4 shows the risk-of-bias assessments for the individual domains of the Cochrane risk of bias tool. Absence of blinding of participants and personnel (staff) was the most common reason for a high risk of bias.

## Discussion

The aim of this study was to describe the outcomes used in recent RCTs evaluating clinical nutrition interventions in critically ill patients. The most commonly used primary outcome was the complication rate, which was related to both the efficacy (infectious complications) and tolerance (metabolic complications and feeding intolerance) of the nutritional intervention. Mortality and duration of organ dysfunction were the primary outcomes in a quarter of the trials, whereas functional outcomes were only very rarely used.

The complication rate may seem relevant to an assessment of both efficacy - as adequate nutrition is believed to decrease the risk of infectious complications - and good tolerance with fewer metabolic and feeding complications. However, the complication rate reflects only limited and expected effects of nutritional interventions. In addition, the definition of complications is not standardised.

In the most recent and largest RCTs, mortality was widely used as the primary outcome. Mortality is an objective outcome that is easy to collect, free of interpretation bias, and clinically relevant. These advantages may increase the chances of obtaining funding. However, the use of six different mortality time points in the RCTs included in our analysis hampers comparisons across studies. Furthermore, mortality as the primary outcome is not necessarily associated with quality of care [13]. A single and usually short-term nutritional intervention may be unlikely to improve long-term mortality in patients with severe critical illness. As with many RCTs in critically ill patients, those using mortality as the primary outcome showed no significant differences between groups. One possible explanation is that a nutritional intervention may have benefits in some patients but cause harm in others. Another is that predicted mortality varies widely across ICU patients included in RCTs.

Mortality and ICU and hospital length of stay are often used as outcomes in studies of critically ill patients. However, survivors of critical illness may experience profound physical, cognitive, and psychological impairments that may alter their long-term quality of life [14]. Outcomes other than mortality that are likely to be important for patients, i.e., patient-important outcomes, were rarely used in the RCTs included in our review, although they allow a more accurate estimate of the long-term health burden of critical illness [15]. Assessing functional outcomes has long been viewed as challenging, due to differences across patients in baseline parameters (before ICU admission) and to the absence of validated tools for evaluating post-ICU function. The emerging concept of frailty in critically ill patients refers to a limitation of physical and cognitive reserve that impairs the ability to cope with stressors, thereby increasing the risk of adverse events during the ICU stay. The frailty score categorizes patients according to their pre-ICU status and helps to assess the risk of lower survival and of poorer intermediate and long-term functional outcomes.

Considering functional outcomes in future trials may better define the benefits of nutritional interventions. Muscle strength can be assessed using dynamometry or the Medical Research Council Scale [16] and muscle mass by ultrasound or computed tomography. Tools available for assessing physical function include the 6-min walking test, Chelsea Critical Care Physical Assessment Tool (CPAx) [17], and Functional Status Score for the Intensive Care Unit (FFS-ICU) [18, 19]. Functional self-sufficiency can be evaluated using instruments such as the Barthel Activities of Daily Living Index [20] or the Functional Independence Measure [21]. Quality-of-life scales may also be useful.

The RCTs included in our study used a broad diversity of outcomes, with six different categories, each containing several outcomes. Furthermore, one out of five RCT reports did not specify whether the outcomes were primary or secondary. This heterogeneity may reflect differences in study populations and interventions. Nutritional interventions cover a vast spectrum of treatments that differ in factors such as the energy target, composition and route of administration of the preparations, and timing of delivery. This last point is of special importance. Although the acute and chronic/recovery phases are challenging to define in clinical practice, similar nutritional interventions probably achieve different goals according to the individual patient’s metabolic response to the insult. Thus, the wide diversity in outcome measures identified in the present study can be ascribed to differences in research objectives, patient populations, and interventions.

Nevertheless, this heterogeneity of outcomes may complicate the interpretation and comparison of results across studies, thereby limiting the ability to conduct valid meta-analyses. It therefore indicates a need to develop a consensus about a minimum core outcome set (COS) for critical-care nutrition trials, to improve the consistency of outcome selection and measurement. The wide spectrum of outcomes identified in our study shows that much work will be needed to obtain such a consensus. In the field of paediatric critical care, the Core Outcome Measures in Effectiveness Trials (COMET) initiative is working towards identifying core outcome measures [22]. As most studies in paediatric critical care are small and conducted at a single centre, the ability to combine data sets for analysis is of paramount importance. The COMET initiative will systematically describe the outcome measures used in these paediatric studies then conduct an international Delphi study to agree on a standard set of core outcomes for future trials. Our review was conducted as a preliminary to developing a set of valid, reliable, and feasible core outcomes for adults, with the goal of improving the quality of research results by decreasing study heterogeneity and outcome-reporting bias and by increasing the statistical power of meta-analyses. The development of a COS is supported by the COMET group and has been endorsed by the Cochrane Library, World Health Organisation, and Grading of Recommendations Assessment, Development and Evaluation (GRADE) working group [23, 24]. The use of a COS for clinical nutrition research in ICUs may help to compare nutritional strategies, effectively pool data from different studies on the same condition, and encourage more complete reporting of outcomes.

When developing and applying a COS for critically ill patients and for specific nutritional interventions, it is important to consider the setting. For example, studies of nutrition in the ICU may include death as an essential outcome. However, as overall mortality declines with advances in intensive care, mortality may be low in all groups and therefore less relevant to comparisons of treatment strategies. Other outcomes, such as quality of life at discharge and muscle strength, may be more relevant. Therefore, once developed, the COS should be reappraised continually in the light of changes in practices, techniques, and patient outcomes. Furthermore, and importantly, the COS should be viewed as the minimum set of parameters that must be measured and reported. If parameters not included in the COS are relevant in the setting of a given study, they should also be measured and recorded. Such additional parameters may be particularly likely to exist in critical care, given the complexity of the conditions seen in ICU patients.

To harmonise outcome selection for studies of nutrition in the ICU, we are planning an international Delphi consensus process in which experts will be asked about outcome assessments appropriate for different study objectives, and patients will be asked about the outcomes they believe are most meaningful. Importantly, the present study did not focus on heterogeneity in outcome definitions but, instead, on the diversity of outcomes across trials. The main source of bias in the RCTs included in our study was absence of blinding of the patients and staff.

A potential limitation is that our study was confined to RCTs published in or after 2000. However, our goal was to capture the most recent practices in RCT design. In addition, very few RCTs published before 2000 focussed on nutrition in ICU patients. Our objective was to obtain an overview of the outcomes used and their definitions, regardless of the trial results, which our study was not designed to evaluate.

## Conclusion

The work reported here is the first systematic review providing detailed information on the objectives and outcomes of RCTs assessing nutritional interventions in critically ill patients. The results indicate considerable heterogeneity in selected outcomes. This heterogeneity can be ascribed in part to the diversity of the study interventions (in terms of composition, energy targets, route, and timing) and study populations.

The results of our systematic review have implications for designing future RCTs of nutritional interventions in the ICU and may serve as a first step towards developing a COS including patient-important outcomes for use in future RCTs [15, 24].

## Additional file

Additional file 1: PubMed search, ClinicalTrials.gov and Cochrane library search strategies. (DOCX 26 kb)

Additional file 2: Characteristics of included published studies (1–142) and protocols of future ongoing trials registered on ClinicalTrials.gov (143 to 170). (DOCX 67 kb)

## Abbreviations

COMET: Core Outcome Measures in Effectiveness Trials
COS: Core outcome set
EN: Enteral nutrition
PN: Parental nutrition
PRISMA: Preferred reporting items for systematic reviews and meta-analyses
