# Supplementary material for: Outcomes used in randomised controlled trials of nutrition in the critically ill: a systematic review
Source: Crit Care. 2019 Jan 14;23:12. doi: 10.1186/s13054-018-2303-7 (PMC6332589; doi:10.1186/s13054-018-2303-7)
Supplement: Supplementary file 1 — PubMed search, ClinicalTrials.gov and Cochrane library search strategies. (DOCX 26 kb) [file 13054_2018_2303_MOESM1_ESM.docx]

## Additional file 1

**PubMed search strategy (n=433)**

(nutrition[Title/Abstract] OR feeding[Title/Abstract] OR alimentation[Title/Abstract] OR protein-calorie[Title/Abstract] OR Nutritional support[Title/Abstract])) AND (Critical care[Title/Abstract] OR Critically ill[Title/Abstract] OR Intensive care[Title/Abstract] OR ICU[Title/Abstract] OR Critical illness[Title/Abstract]) AND all adult[filter] NOT infant[filter])) AND (Randomized controlled trial[Publication Type] OR Controlled clinical trial[Publication Type] OR Randomized[Title/Abstract] OR Trial[Title/Abstract]))) NOT review[Filter]) AND Humans[MeSH] AND (("2000/01/01"[PDat]: "2018/08/31"[PDat] ) AND Humans[MeSH])

**Clinicaltrial.gov search strategy (n=274)**

Interventional studies | (nutrition OR feeding OR Protein-calorie OR Nutritional support) AND (Critical care OR Critically ill OR Intensive care OR ICU OR Critical illness) AND Randomized | Adult, Older adult | Start date from 01/01/2000 to 08/31/2018

**Cochrane library search strategy (n=428)**

Ti;ab;kw: nutrition OR feeding OR "protein-calorie" OR "Nutritional support"

AND

Ti;ab;kw: "Critical care" OR "Critically ill" OR "Intensive care" OR "ICU" OR "Critical illness"

AND

Pt: "randomized control trial"
