# Supplementary material for: Outcomes used in randomised controlled trials of nutrition in the critically ill: a systematic review
Source: Crit Care. 2019 Jan 14;23:12. doi: 10.1186/s13054-018-2303-7 (PMC6332589; doi:10.1186/s13054-018-2303-7)
Supplement: Supplementary file 2 — Characteristics of included published studies (1–142) and protocols of future ongoing trials registered on ClinicalTrials.gov (143 to 170). (DOCX 67 kb) [file 13054_2018_2303_MOESM2_ESM.docx]

**Characteristics of included published studies (1 to 142) and protocols of future ongoing trials registered on Clinicaltrials.gov (143 to 170)**

|  | **Title** | **Journal** | **Authors** | **Year of Publication** |
| --- | --- | --- | --- | --- |
| 1 | Effects of pectin on diarrhea in critically ill tube-fed patients receiving antibiotics | American Journal Of Critical Care | A. Schultz | 2000 |
| 2 | Enteral feeding with a solution enriched with antioxidant vitamins A, C, and E enhances the resistance to oxidative stress | Critical Care Medicine | J C. Preiser and al. | 2000 |
| 3 | Total parenteral nutrition enriched with arginine and glutamate generates glutamine and limits protein catabolism in surgical patients hospitalized in intensive care units | Critical Care Medicine | M P. Bérard and al. | 2000 |
| 4 | Early versus delayed feeding with an immune-enhancing diet in patients with severe head injuries | Journal Of Parenteral And Enteral Nutrition | G. Minard and al. | 2000 |
| 5 | Nitrogen sparing e¡ect of structured triglycerides containing both medium-and long-chain fatt acids in critically ill patients; a double blind randomized controlled trial | Clinical Nutrition | B F. Lindgren and al. | 2001 |
| 6 | Enteral feeding in the critically ill: comparison between the supine and prone positions. A prospective cross-over study in mechanically ventilated patients | Critical Care | P HJ. Van der Voort and D F. Zandstra | 2001 |
| 7 | Effect of postpyloric feeding on gastroesophageal regurgitation and pulmonary microaspiration: Results of a randomized controlled trial | Critical Care Medicine | D K. Heyland and al. | 2001 |
| 8 | Effects of intravenous fat emulsions on lung function in patients with acute respiratory distress syndrome or sepsis | Critical Care Medicine | U. Suchner and al. | 2001 |
| 9 | Glutamine administration reduces Gram-negative bacteremia in severely burned patients: A prospective, randomized, double-blind trial versus isonitrogenous control | Critical Care Medicine | P E. Wischmeyer and al. | 2001 |
| 10 | Early enteral nutrition in critically iII patients with a high-protein diet enriched with arginine, fiber, and antioxidants compared with a standard high-protein diet. The effect on nosocomial infections and outcome | Journal Of Parenteral And Enteral Nutrition | T. Caparrós and al. | 2001 |
| 11 | Metabolic effects of arginine addition to the enteral feeding of critically iII patients | Journal Of Parenteral And Enteral Nutrition | J C. Preiser and al. | 2001 |
| 12 | Influence of polymeric enteral nutrition supplemented with different doses of glutamine on gut permeability in critically ill patients | Nutrition | N. Velasco and al. | 2001 |
| 13 | The local vascular tolerance to an intravenous infusion of a concentrated glutamine solution in ICU patients | Clinical Nutrition | A. Berg et al. | 2002 |
| 14 | Gastric versus small-bowel tube feeding in the intensive care unit: A prospective comparison of efficacy | Critical Care Medicine | D A. Neumann and al. | 2002 |
| 15 | Multicenter, prospective, randomized, single-blind study comparing the efficacy and gastrointestinal complications of early jejunal feeding with early gastric feeding in critically ill patients | Critical Care Medicine | J. Montejo and al. | 2002 |
| 16 | Parenteral L-alanyl-L-glutamine improves 6-month outcome in critically ill patients | Critical Care Medicine | C. Goeters and al. | 2002 |
| 17 | Randomized comparison of nasojejunal and nasogastric feeding in critically ill patients | Critical Care Medicine | A R. Davies and al. | 2002 |
| 18 | A randomized prospective trial of immediate vs. next-day feeding after percutaneous endoscopic gastrostomy in intensive care patients | Intensive Care Medicine | J. Stein et al. | 2002 |
| 19 | Early versus late enteral feeding of mechanically ventilated patients: results of a clinical trial | Journal Of Parenteral And Enteral Nutrition | E H. Ibrahim and al. | 2002 |
| 20 | Clinical and metabolic effects of two lipid emulsions on the parenteral nutrition of septic patients | Nutrition | J. Garnacho-Montero and al. | 2002 |
| 21 | Effect of a Glutamine-Enriched Enteral diet on intestinal permeability and infectious morbidity at 28 days in critically ill patients with systemic inflammatory response syndrome: A randomized, single-blind, prospective, multicenter study | Nutrition | R. Conejero and al. | 2002 |
| 22 | Infection, multiple organ failure, and survival in the intensive care unit: influence of glutamine-supplemented parenteral nutrition on acquired infection | Nutrition | R D. Griffiths and al. | 2002 |
| 23 | Enteral nutrition with eicosapentaenoic acid, -linolenic acid, and antioxidants reduces alveolar inflammatory mediators and protein influx in patients with acute respiratory distress syndrom | Critical Care Medicine | E R. Pacht and al. | 2003 |
| 24 | A prospective randomized trial of enteral glutamine in critical illness | Intensive Care Medicine | J. Hall et al. | 2003 |
| 25 | Early enteral immunonutrition in patients with severe sepsis | Intensive Care Medicine | G. Bertolini and al. | 2003 |
| 26 | ω-3 vs. ω-6 lipid emulsions exert differential influence on neutrophils in septic shock patients: impact on plasma fatty acids and lipid mediator generation | Intensive Care Medicine | K. Mayer and al. | 2003 |
| 27 | Multicentre, cluster-randomized clinical trial of algorithms for critical-care enteral and parenteral therapy (ACCEPT) | Canadian Medical Association Journal | C M. Martin and al. | 2004 |
| 28 | Control of diarrhea by fiber-enriched diet in ICU patients on enteral nutrition: a prospective randomized controlled trial | Clinical Nutrition | T A. Rushdi et al. | 2004 |
| 29 | Influence of synbiotic containing Lactobacillus acidophilus La5, Bifidobacterium lactis Bb 12, Streptococcus thermophilus, Lactobacillus bulgaricus and oligofructose on gut barrier function and sepsis in critically ill patients: a randomised controlled trial | Clinical Nutrition | P. Jain and al. | 2004 |
| 30 | Is early enteral nutrition a risk factor for gastric intolerance 29and pneumonia? | Clinical Nutrition | L. Kompan et al. | 2004 |
| 31 | L-Alanyl-L-glutamine-supplemented parenteral nutrition improves infectious morbidity in secondary peritonitis | Clinical Nutrition | C. Fuentes-Orozco and al. | 2004 |
| 32 | The effects of standard and branched chain amino acid enriched solutions on thermogenesis and energy expenditure in unconscious intensive care patients | Clinical Nutrition | I. Cankayali and al. | 2004 |
| 33 | Benefits of early enteral nutrition with glutamine and probiotics in brain injury patients | Clinical Science | I. Arruda et Aguilar-Nascimento | 2004 |
| 34 | The beneficial effects of antioxidant supplementation in enteral feeding in critically ill patients: A prospective, randomized, double-blind, placebo-controlled trial | Critical Care And Trauma | E. Crimi and al. | 2004 |
| 35 | A randomized trial of endoscopic and fluoroscopic placement of postpyloric feeding tubes in critically ill patients | Journal Of Parenteral And Enteral Nutrition | J A. Foote et al. | 2004 |
| 36 | Percutaneous radiologic gastrostomy versus nasogastric tube in critically ill patients | Clinical Nutrition | P-M. Roy and al. | 2005 |
| 37 | Clinical outcome of immunonutrition in a heterogeneous intensive care population | Intensive Care Medicine | H. Kieft et al. | 2005 |
| 38 | Glucose-lipid ratio is a determinant of nitrogen balance during total parenteral nutrition in critically ill patients: a prospective, randomized, multicenter blind trial with an intention-to-treat analysis | Intensive Care Medicine | P. Boulétreau and al. | 2005 |
| 39 | Olive oil based nutrition in multiple trauma patients: a pilot study | Intensive Care Medicine | G. Huschak and al. | 2005 |
| 40 | Parenteral glutamine increases serum heat shock protein 70 in critically ill patients | Intensive Care Medicine | T. Ziegler and al. | 2005 |
| 41 | Albumin administration improves organ function in critically ill hypoalbuminemic patients: A prospective, randomized, controlled, pilot stud | Critical Care Medicine | M J. Dubois and al. | 2006 |
| 42 | Effects of enteral feeding with eicosapentaenoic acid, -linolenicacid, and antioxidants in mechanically ventilated patients with severe sepsis and septic shock | Critical Care Medicine | A. Pontes-Arruda and al. | 2006 |
| 43 | L-alanyl-L-glutamine dipeptide–supplemented total parenteral nutrition reduces infectious complications and glucose intolerance in critically ill patients: The French controlled, randomized, double-blind, multicenter study | Critical Care Medicine | P. Déchelotte and al. | 2006 |
| 44 | Metabolic effects of parenteral nutrition enriched with n-3 polyunsaturated fatty acids in critically ill patients | Clinical Nutrition | L. Tappy and al. | 2006 |
| 45 | Early enteral immunonutrition vs. parenteral nutrition in critically ill patients without severe sepsis: a randomized clinical trial | Intensive Care Medicine | D. Radrizzani et al. | 2006 |
| 46 | Analysis of sites of bacterial contamination in an enteral feeding system | Journal Of Parenteral And Enteral Nutrition | M H. Mathus-Vliegen et al. | 2006 |
| 47 | A diet enriched in eicosapentanoic acid, gamma-linolenic acid and antioxidants in the prevention of new pressure ulcer formation in critically ill patients with acute lung injury: A randomized, prospective, controlled study | Clinical Nutrition | M. Theilla and al. | 2007 |
| 48 | Effect of an enteral diet supplemented with a specific blend of amino acid on plasma and muscle protein synthesis in ICU patients | Clinical Nutrition | O. Mansoor and l. | 2007 |
| 49 | Synbiotics, Prebiotics, Glutamine, or peptide in early enteral nutrition: A randomized study in trauma patients | Journal Of Parenteral And Enteral Nutrition | A. Spindler-Vesel and al. | 2007 |
| 50 | Prospective randomized control trial of intermittent versus continuous gastric feeds for critically ill trauma patient | The Journal Of Trauma | J B A. MacLeod and al. | 2007 |
| 51 | Metabolic effects of enteral versus parenteral alanyl-glutamine dipeptide administration in critically ill patients receiving enteral feeding: a pilot study | Clinical Nutrition | M. Luo and al. | 2008 |
| 52 | Early enteral supplementation with key pharmaconutrients improves sequential organ failure assessment score in critically ill patients with sepsis: Outcome of a randomized, controlled, double-blind trial | Critical Care Medicine | J B. Richard and al. | 2008 |
| 53 | Oral probiotic and prevention of Pseudomonas aeruginosa infections: a randomized, double-blind, placebo-controlled pilot study in intensive care unit patients | Critical Care | C. Forestier and al. | 2008 |
| 54 | The impact of delaying enteral feeding on gastric emptying, plasma cholecystokinin, and peptide YY concentrations in critically ill patient | Critical Care Medicine | N Q. Nguyen and al. | 2008 |
| 55 | Fish oil supplementation in the parenteral nutrition of critically ill medical patients: a randomised controlled trial | Intensive Care Medicine | S. Friesecke and al. | 2008 |
| 56 | Initial efficacy and tolerability of early enteral nutrition with immediate or gradual introduction in intubated patients | Intensive Care Medicine | A. Desachy and al. | 2008 |
| 57 | Effect of evidence-based feeding guidelines on mortality of critically ill adults | Jama | G S. Doig and al. | 2008 |
| 58 | Efficacy of parenteral nutrition supplemented with glutamine Dipeptide to decrease hospital infections in critically ill surgical patients | Journal Of Parenteral And Enteral Nutrition | F. Estívariz an al. | 2008 |
| 59 | Enteral glutamine during active shock resuscitation is safe and Enhances tolerance of enteral feeding | Journal Of Parenteral And Enteral Nutrition | M. McQuiggan and al. | 2008 |
| 60 | Early hypocaloric enteral nutritional supplementation in acute organophosphate poisoning – a prospective randomized trial | Clinical Toxicology | V. Moses and al. | 2009 |
| 61 | A randomised controlled comparison of early post-pyloric versus early gastric feeding to meet nutritional targets in ventilated intensive care patients | Critical Care | H. White and al. | 2009 |
| 62 | A randomised controlled comparison of early post-pyloric versus early gastric feeding to meet nutritional targets in ventilated intensive care patients | Critical Care Medicine | H. White and al. | 2009 |
| 63 | Duodenal versus gastric feeding in medical intensive care unit patients: A prospective, randomized, clinical study | Critical Care Medicine | C-W. Hsu and al. | 2009 |
| 64 | Decrease in frequency of liquid stool in enterally fed critically ill patients given the multispecies probiotic: a pilot trial | American Journal Of Critical Care | T J. Frohmader et al. | 2010 |
| 65 | Mixed fibers diet in surgical ICU septic patients | Asia Pacific Journal Of Clinical Nutrition | K. Chittawatanarat and al. | 2010 |
| 66 | Randomized clinical trial randomized clinical trial of gut-specific nutrients in critically ill surgical patients | British Journal Of Surgery | M. Gatt and J. MacFie | 2010 |
| 67 | Effects of a fish oil containing lipid emulsion on plasma phospholipid fatty acids, inflammatory markers, and clinical outcomes in septic patients: a randomized, controlled clinical trial. | Critical Care | V M. Barbosa et al. | 2010 |
| 68 | Lack of effect of glutamine administration to boost the innate immune system response in trauma patients in the intensive care unit | Critical Care Medicine | J. Pérez-Bárcena et al. | 2010 |
| 69 | Gastric residual volume during enteral nutrition in ICU patients: the REGANE study | Intensive Care Medicine | JC. Montejo and al. | 2010 |
| 70 | Gastric versus transpyloric feeding in severe traumatic brain injury: a prospective, randomized trial | Intensive Care Medicine | J. Acosta-Escribano and al. | 2010 |
| 71 | Ginger extract reduces delayed gastric emptying and nosocomial pneumonia in adult respiratory distress syndrome patients hospitalized in an intensive care unit | Journal Of Critical Care | Z V. Shariatpanahi AND al. | 2010 |
| 72 | Safer glycemic control using isomaltulose-based enteral formula: A pilot randomized crossover trial | Journal Of Critical Care | M. Egi et al. | 2010 |
| 73 | Scandinavian glutamine trial: a pragmatic multi-centre randomised clinical trial of intensive care unit patients | Acta Anaesthesiologica Scandinavica | J. Wernerman and al. | 2011 |
| 74 | Randomised trial of glutamine, selenium, or both, to supplement parenteral nutrition for critically ill patients | British Medical Journal | P. Andrews and al. | 2011 |
| 75 | Short-term effects of high-dose oral vitamin D3 in critically ill vitamin D deficient patients: a randomized, double-blind, placebo-controlled pilot study | Critical Care | K. Amrein and al. | 2011 |
| 76 | Effect of an enteral diet enriched with eicosapentaenoic acid, gamma-linolenic acid and anti-oxidants on the outcome of mechanically ventilated, critically ill, septic patients | Clinical Nutrition | T. Grau-Carmona and al. | 2011 |
| 77 | Tolerability and efficacy of a low-volume enteral supplement containing key nutrients in the critically ill | Clinical Nutrition | A. Schneider et al. | 2011 |
| 78 | The impact of L-alanyl-L-glutamine dipeptide supplemented total parenteral nutrition on clinical outcome in critically patients | Clinical Nutrition Espen | N. Cekmen and al. | 2011 |
| 79 | Effects of probiotics on serum levels of Th1/Th2 cytokine and clinical outcomes in severe traumatic brain-injured patients: a prospective randomized pilot study | Critical Care | M. Tan et al. | 2011 |
| 80 | A randomized trial of initial trophic versus full-energy enteral nutrition in mechanically ventilated patients with acute respiratory failure | Critical Care Medicine | T W. Rice | 2011 |
| 81 | Jejunal tube placement in critically ill patients: A prospective, randomized trial comparing the endoscopic technique with the electromagnetically visualized method | Critical Care Medicine | U. Holzinger and al. | 2011 |
| 82 | Poor validity of residual volumes as a marker for risk of aspiration in critically ill patient | Critical Care Medicine | S A. MCClave | 2011 |
| 83 | The effect of L-alanyl-L-glutamine dipeptide supplemented total parenteral nutrition on infectious morbidity and insulin sensitivity in critically ill patients | Critical Care Medicine | T. Grau and al. | 2011 |
| 84 | The tight calorie control study (TICACOS): a prospective, randomized, controlled pilot study of nutritional support in critically ill patients | Intensive Care Medicine | P. Singer and al. | 2011 |
| 85 | Early parenteral nutrition in critically ill patients with short-term relative contraindications to early enteral nutrition, A randomized controlled trial | Jama | G S. Doig and al. | 2011 |
| 86 | Enteral omega-3 fatty acid, γ-Linolenic acid, and antioxidant supplementation in acute lung injury | Jama | T. Rice and al. | 2011 |
| 87 | Lactobacillus GG as treatment for diarrhea during enteral feeding in critical illness: randomized controlled trial | Journal Of Parenteral And Enteral Nutrition | S. Ferrie and M. Daley | 2011 |
| 88 | Effects of an omega-3 fatty acid-enriched lipid emulsion on eicosanoid synthesis in acute respiratory distress syndrome (ARDS): A prospective, randomized, double-blind, parallel group study | Nutrition And Metabolism | J. Sabater and al. | 2011 |
| 89 | Effect of enteral versus parenteral nutrition on outcome of medical patients requiring mechanical ventilation | Nutrition In Clinical Practice | N D. Altintas | 2011 |
| 90 | Permissive underfeeding and intensive insulin therapy in critically ill patients: a randomized controlled trial | The American Journal Of Clinical Nutrition | Y M. Arabi and al. | 2011 |
| 91 | Early versus late parenteral nutrition in critically ill adults | The New England Journal Of Medicine | M P. Casaer and al. | 2011 |
| 92 | Impact of a nutritional formula enriched in fish oil and micronutrients on pressure ulcers in critical care patients | American Journal Of Critical Care | M. Theilla and al. | 2012 |
| 93 | Enteral n-3 fatty acids and micronutrients enhance percentage of positive neutrophil and lymphocyte adhesion molecules: a potential mediator of pressure ulcer healing in critically ill patients | British Journal Of Nutrition | M. Theilla and al. | 2012 |
| 94 | A double-blind, randomized clinical trial comparing soybean oil–based versus olive oil–based lipid emulsions in adult medical–surgical intensive care unit patients requiring parenteral nutrition | Critical Care Medicine | G E. Umpierrez and al. | 2012 |
| 95 | A multicenter, randomized controlled trial comparing early nasojejunal with nasogastric nutrition in critical illness | Critical Care Medicine | A R. Davies and al. | 2012 |
| 96 | Initial trophic vs full enteral feeding in patients with acute lung injury the EDEN randomized trial | Jama | T W. Rice and al. | 2012 |
| 97 | Severity of illness influences the efficacy of enteral feeding route on clinical outcomes in patients with critical illness | Journal Of Academy Of Nutrition And Dietetics | H-H. Huang and al. | 2012 |
| 98 | Influence of parenteral nutrition delivery system on the development of blood stream infections in critically ill patients: An international, multicenter, prospective open-label, controlled study - EPICOS study | Journal Of Parenteral And Enteral Nutrition | A. Pontes-Arruda and al. | 2012 |
| 99 | Markers of inflammation and coagulation may be modulated by enteral feeding strategy | Journal Of Parenteral And Enteral Nutrition | J. Bastarache and al. | 2012 |
| 100 | Enteral nutrition supplemented with L-glutamine in patients with systemic inflammatory response syndrome due to pulmonary infection | Nutrition | A A. Cavalcante and al. | 2012 |
| 101 | Effects of fish oil on inflammatory modulation in surgical intensive care unit patients | Nutrition In Clinical Practice | Y Y. Han and al. | 2012 |
| 102 | Early initiation of enteral nutrition improves outcomes in burn disease | Asia Pacific Journal Of Clinical Nutrition | V. Kovacic Vicic and al. | 2013 |
| 103 | Impact of early versus late enteral nutrition on cell mediated immunity and its relationship with glucagon like peptide-1 in intensive care unit patients: a prospective study | Critical Care | O. Bakiner et al. | 2013 |
| 104 | Enhanced protein-energy provision via the enteral route feeding protocol in critically ill patients: Results of a cluster randomized trial | Critical Care Medicine | D K. Heyland and al. | 2013 |
| 105 | Hyperproteic hypocaloric enteral nutrition in the critically ill patient: A randomized controlled clinical trial | Indian Journal Of Critical Care Medicine | S J. Rugeles and al. | 2013 |
| 106 | Effect of not monitoring residual gastric volume on risk of ventilator-associated pneumonia in adults receiving mechanical ventilation and early enteral feeding | Jama | J. Reignier and al. | 2013 |
| 107 | Effect of enteral feeding with ginger extract in acute respiratory distress syndrome | Journal Of Critical Care | Z. Vahdat Shariatpanahi and al. | 2013 |
| 108 | Optimisation of energy provision with supplemental parenteral nutrition in critically ill patients: a randomised controlled clinical trial | Lancet | C P. Heidegger and al. | 2013 |
| 109 | A randomized trial of glutamine and antioxidants in critically ill patients | The New England Journal Of Medicine | D. Heyland and al. | 2013 |
| 110 | Effects of early enteral nutrition on immune function of severe acute pancreatitis patients | World Journal Of Gastroenterology | J-K. Sun and al. | 2013 |
| 111 | A randomized, double-blind, placebo-controlled trial of caspofung in prophylaxis followed by preemptive therapy for invasive candidiasis in high-risk adults in the critical care setting | Clinical Infectious Diseases | L. Ostrosky-Zeichner and al. | 2014 |
| 112 | Endogenous glutamine production in critically ill patients: the effect of exogenous glutamine supplementation | Critical Care | M. Mori and al. | 2014 |
| 113 | A randomized trial of intravenous glutamine supplementation in trauma ICU patients | Intensive Care Medicine | J. Pérez-Bárcena et al. | 2014 |
| 114 | High-protein enteral nutrition enriched with immune-modulating nutrients vs standard high-protein enteral nutrition and nosocomial infections in the ICU | Jama | A R H. Zanten and al. | 2014 |
| 115 | Effects of lipid emulsions in parenteral nutrition of esophageal cancer surgical patients receiving enteral nutrition: A comparative analysis | Nutrients | W-P. Wang and al. | 2014 |
| 116 | Hypocaloric compared with eucaloric nutritional support and its effect on infection rates in a surgical intensive care unit: a randomized controlled trial | The American Journal Of Clinical Nutrition | E J. Charles | 2014 |
| 117 | Use of a concentrated enteral nutrition solution to increase calorie delivery to critically ill patients: a randomized, double-blind, clinical trial | The American Journal Of Clinical Nutrition | S. Peake et al. | 2014 |
| 118 | Trial of the route of early nutritional support in critically ill adults | The New England Journal Of Medicine | S E. Harvey et al. | 2014 |
| 119 | Permissive Underfeeding or Standard Enteral Feeding in Critically Ill Adults | The American Journal Of Clinical Nutrition | Y M. Arabi and al. | 2015 |
| 120 | The influence of parenteral glutamine supplementation on glucose homeostasis in critically ill polytrauma patients: A randomized-controlled clinical study | Clinical Nutrition | I M. Grintescu et al. | 2015 |
| 121 | Diabetes-specific enteral nutrition formula in hyperglycemic, mechanically ventilated, critically ill patients: a prospective, open-label, blind-randomized, multicenter study | Critical Care Medicine | A. Mesejo and al. | 2015 |
| 122 | The impact of abdominal massage administered to intubated and enterally fed patients on the development of ventilator-associated pneumonia: A randomized controlled study | International Journal Of Nursing Studies | B B. Kahraman and L. Ozdemir | 2015 |
| 123 | Effect of enteral diet enriched with eicosapentaenoic acid, gamma-linolenic acid, and antioxidants in patients with sepsis-induced acute respiratory distress syndrome | Journal Of Intensive Care | K. Shirai and al. | 2015 |
| 124 | Intensive nutrition in acue lung injury: A Clinical Trial (INTACT) | Journal Of Parenteral And Enteral Nutrition | C A. Braunschweig and al. | 2015 |
| 125 | Efficacy and Safety of Glutamine-supplemented Parenteral Nutrition in Surgical ICU Patients: An American Multicenter Randomized Controlled Trial. | Annals Of Surgery | T R. Ziegler and al. | 2016 |
| 126 | A randomised controlled feasibility and proof-of-concept trial in delayed gastric emptying when metoclopramide fails: We should revisit nasointestinal feeding versus dual prokinetic treatment: Achieving goal nutrition in critical illness and delayed gastric emptying: Trial of nasointestinal feeding versus nasogastric feeding plus prokinetics. | Clinical Nutrition Espen | S J. Taylor and al. | 2016 |
| 127 | High-protein hypocaloric vs normocaloric enteral nutrition in critically ill patients: A randomized clinical trial | Journal Of Critical Care | S. Rugeles and al. | 2016 |
| 128 | Effect of probiotics on the incidence of ventilator-associated pneumonia in critically ill patients: a randomized controlled multicenter trial. | Intensive Care Medicine | J. Zeng and al. | 2016 |
| 129 | Evaluation of the effect on patient parameters of not monitoring gastric residual volume in intensive care patients on a mechanical ventilator receiving enteral feeding: A randomized clinical trial | Journal Of Critical Care | N. Ozen and al. | 2016 |
| 130 | Microbial cell preparation in enteral feeding in critically ill patients: A randomized, double-blind, placebo-controlled clinical trial. | Journal Of Critical Care | AA. Malik and al. | 2016 |
| 131 | Hypocaloric vs normocaloric nutrition in critically Ill patients: A prospective randomized pilot trial. | Journal Of Parenteral And Enteral Nutrition | S. Petros and al. | 2016 |
| 132 | Protein Requirements in the Critically Ill: A Randomized Controlled Trial Using Parenteral Nutrition. | Journal Of Parenteral And Enteral Nutrition | S. Ferrie and al. | 2016 |
| 133 | Randomized Controlled Trial Assessing the Feasibility of Shortened Fasts in Intubated ICU Patients Undergoing Tracheotomy. | Otolaryngol Head Neck Surg | N. Gonik and al. | 2016 |
| 134 | Effects of immunonutrition on biomarkers in traumatic brain injury patients in Malaysia: a prospective randomized controlled trial. | Bmc Anesthesiology | VRH. Rai and al. | 2017 |
| 135 | Pilot study evaluating the efficacy, tolerance and safety of a peptide-based enteral formula versus a high protein enteral formula in multiple ICU settings (medical, surgical, cardiothoracic) | Clinical Nutrition | D S. Seres and P R. Ippolito | 2017 |
| 136 | A randomized controlled pilot study to evaluate the effect of an enteral formulation designed to improve gastrointestinal tolerance in the critically ill patient-the SPIRIT trial. | Critical Care | SM. Jakob and al. | 2017 |
| 137 | A randomized trial of supplemental parenteral nutrition in underweight and overweight critically ill patients: the TOP-UP pilot trial | Critical Care | P E. Wischmeyer and al. | 2017 |
| 138 | Early goal-directed nutrition versus standard of care in adult intensive care patients: the single-centre, randomised, outcome assessor-blinded EAT-ICU trial. | Intensive Care Medicine | MJ. Allingstrup and al. | 2017 |
| 139 | Fat-Modified Enteral Formula Improves Feeding Tolerance in Critically Ill Patients: A Multicenter, Single-Blind, Randomized Controlled Trial. | Journal Of Parenteral And Enteral Nutrition | C. Qiu and al. | 2017 |
| 140 | Standard vs. Calorie-Dense Immune Nutrition in Haemodynamically Compromised Cardiac Patients: A Prospective Randomized Controlled Pilot Study | Nutrients | S. Efremov and al. | 2017 |
| 141 | Enteral nutrition as stress ulcer prophylaxis in critically ill patients: A randomized controlled exploratory study. | Journal Of Critical Care | K. El-Kersh and al. | 2018 |
| 142 | Enteral versus parenteral early nutrition in ventilated adults with shock: a randomised, controlled, multicentre, open-label, parallel-group study (NUTRIREA-2) | Lancet | J.Reignier and al. | 2018 |

| **Reference** | **Title** | **Protocols** | **Authors** | **Recruitment status** |
| --- | --- | --- | --- | --- |
| 143 | High Protein Intake and Early Exercise in Adult Intensive Care Patients | CLINICALTRIAL.GOV | W S. MONTENEGRO and al. | Not yet recruiting |
| 144 | Intensive Nutrition in Critically Ill Adults | CLINICALTRIAL.GOV | E. Ridley and al. | Not yet recruiting |
| 145 | Promotion of oesophageal motility to prevent regurgitation and enhance nutrition intake in ICU patients. (PROPEL) | CLINICALTRIAL.GOV | D K. Heyland and al. | Not recruiting |
| 146 | The energy dose study | CLINICALTRIAL.GOV | T R. Ziegler and al. | Not recruiting |
| 147 | Amino Acid nutrition in the critically-ill (AA-ICU) | CLINICALTRIAL.GOV | A. Kristof and al. | Recruiting |
| 148 | Continuous versus intermittent enteral feeding in critically ill patients: a prospective, randomized controlled trial | CLINICALTRIAL.GOV | J. Lee and al. | Recruiting |
| 149 | Efficacy and safety study of a low-carbohydrate tube feed in critically ill patients under insulin therapy | CLINICALTRIAL.GOV | R. Beer and al. | recruiting |
| 150 | Nutrition and Exercise in Critical Illness (NEXIS) | CLINICALTRIAL.GOV | D K. Heyland and al. | Recruiting |
| 151 | The basel enteral high protein study | CLINICALTRIAL.GOV | M. Siegemund and al. | Recruiting |
| 152 | The Effect of Higher Protein Dosing in Critically Ill Patients (EFFORT) | CLINICALTRIAL.GOV | D K. Heyland and al. | Recruiting |
| 153 | The RE-ENERGIZE Study: RandomizEd trial of ENtERal Glutamine to minimIZE Thermal Injury (RE-ENERGIZE) | CLINICALTRIAL.GOV | D K. Heyland and al. | Recruiting |
| 154 | Directed immuno nutrition by L-arginine for critically ill patients (Immunolarg) | CLINICALTRIAL.GOV | J M. Tadie and al. | Completed |
| 155 | Efficacy of nasojejunal enteral feeding in critically ill patients | CLINICALTRIAL.GOV | M. Robertson and al. | Completed |
| 156 | Enteral feeding composition and acute respiratory failure | CLINICALTRIAL.GOV | Z. Vahdat Shariatpanahi and al. | Completed |
| 157 | ICULIP, influence of two lipid emulsions in the nosocomial infection in critical patients (ICULIP) | CLINICALTRIAL.GOV | G. Ahelardo and al. | Completed |
| 158 | Intermittent versus continuous feeding in ICU patient | CLINICALTRIAL.GOV | N. Hart and al. | Completed |
| 159 | Leptin and Ghrelin in ICU | CLINICALTRIAL.GOV | V. Oggur and al. | Completed |
| 160 | Parenteral nutrition with intravenous and oral fish oil for intensive care patients | CLINICALTRIAL.GOV | P. Singer and al. | Completed |
| 161 | Pectin start early enteral nutritional support in critically ill patients | CLINICALTRIAL.GOV | X. Xu and al. | Completed |
| 162 | Probiotics in enteral feeding in critically ill patients | CLINICALTRIAL.GOV | M. Ausama and al. | Completed |
| 163 | Safety and tolerance on lipids of parenteral and enteral nutrition in critically ill patients with liver failure (SELLIFA) | CLINICALTRIAL.GOV | Y. Fleury and al. | Completed |
| 164 | The augmented versus routine approach to giving energy trial (TARGET) | CLINICALTRIAL.GOV | S. Peake et al. | Completed |
| 165 | The effect of prophylactic probiotic Lactobacilli in enteral feeding on nosocomial pneumonia rates in critically ill patients | CLINICALTRIAL.GOV | M. Robertson and al. | Completed |
| 166 | Effect of enteral nutrition support for critically ill patients | CLINICALTRIAL.GOV | M. Luo and al. | Terminated |
| 167 | Enteral glutamine in critical illness | CLINICALTRIAL.GOV | M. DePietro and al. | Terminated |
| 168 | Enteral versus parenteral glutamine supplement | CLINICALTRIAL.GOV | J. Uranjek and al. | Terminated |
| 169 | Study of nutrition in acute pancreatitis (SNAP) | CLINICALTRIAL.GOV | D. Whithcomb and al. | Terminated |
| 170 | The effects of nutritional support of critically ill patients requiring mechanical ventilation | CLINICALTRIAL.GOV | M. Robertson and al. | Terminated |
